# Research on the Preparation and Application of Fixed-Abrasive Tools Based on Solid-Phase Reactions for Sapphire Wafer Lapping and Polishing

**DOI:** 10.3390/mi14091797

**Published:** 2023-09-20

**Authors:** Linlin Cao, Xiaolong Zhou, Yingjie Wang, Zhilun Yang, Duowen Chen, Wei Wei, Kaibao Wang

**Affiliations:** College of Mechanical Engineering, Beihua University, Jilin 132021, China; caolinlin0626@126.com (L.C.);

**Keywords:** sapphire wafer, fixed-abrasive tool, surface roughness, material removal rate, lapping and polishing

## Abstract

Single-crystal sapphire specimen (α-Al_2_O_3_) have been widely applied in the semiconductor industry, microelectronics, and so on. In order to shorten the production time and improve the processing efficiency of sapphire processing, an integrated fixed-abrasive tool (FAT) based on solid-phase reactions is proposed in this article. The optimal FAT composition is determined using a preliminary experiment and orthogonal experiments. The mass fraction of the abrasives is chosen as 55 wt%, and the mass ratio of SiO_2_/Cr_2_O_3_ is 2. Surface roughness *R*_a_ decreased from 580.4 ± 52.7 nm to 8.1 ± 0.7 nm after 150 min, and the average material removal rate was 14.3 ± 1.2 nm/min using the prepared FAT. Furthermore, FAT processing combined with chemical mechanical polishing (CMP) was shortened by 1.5 h compared to the traditional sapphire production process in obtaining undamaged sapphire surfaces with a roughness of *R*_a_ < 0.4 nm, which may have the potential to take the place of the fine lapping and rough polishing process.

## 1. Introduction

Single-crystal sapphire specimen (α-Al_2_O_3_) are currently one of the most widely applied materials in the fields of defense, aerospace, microelectronics, and the medical domain [1,2,3,4] due to their excellent physicochemical properties and material characteristics, such as high hardness (Mohs hardness 9), high melting point (2045 °C), high light transmittance, high chemical stability, and high thermal stability [5,6,7,8,9,10]. Sapphire has not only become the most widely used substrate material for traditional LEDs [11,12,13,14] but also has a large number of applications in mini/micro-LEDs, which are currently very popular. With the rapid development of mini/micro-LEDs, the demand for high-quality sapphire substrates has increased at the same time. However, sapphire has been generally regarded as a typical hard and brittle material, which is difficult to process. In order to achieve thinning processes with higher material removal rates, free boron carbide and diamond abrasive particles with higher hardness than sapphire are selected in the process of wafer thinning and surface finishing. Machining based on mechanical brittleness removal results in deep scratches on the surface of workpieces, which could produce tens of microns of sub-surface damage layers. This form of damage seriously affects the life and properties of optical components [15,16,17]. Fine polishing as the final process is required to obtain an undamaged surface using CMP (chemical mechanical polishing). At this stage, the commercial mass production process generally takes four to six hours using large professional polishing equipment. Due to centrifugal action, a large number of abrasive particles are thrown out of the processing area with the rotation of the polishing plate, resulting in low abrasive utilization, poor processing uniformity, and high consumable costs.

In order to pursue high efficiency manufacturing, the processing methods of the fixed-abrasive tool (FAT) for sapphire wafers have been diversified by researchers. Wang [18,19] carried out scratch experiments on sapphire specimen with different crystal planes, studied the material removal methods in the grinding process, and carried out in-depth research on the material removal mechanism of sapphire precision grinding and the wear detection of the grinding wheel of diamond abrasives. Luo [20] used precision grinding equipment to process different crystal planes of sapphire specimen and found that the material removal rate and surface roughness of different crystal planes are differentiating properties. The main reason for this difference is the different fracture toughness, Young’s modulus, and surface energy. Niu [21] made a FADA gasket using the mixed particle size agglomerated diamond (MAD) abrasive, which is a combination of ultra-fine diamond particles and coarser diamond particles. The significant grinding feature of MAD abrasive particles is that coarser diamond abrasive particles mainly increase grinding efficiency, while ultra-fine diamond abrasive particles improve the surface’s quality. Wu [22,23] tried using chromium trioxide and silicon oxide as abrasive materials to prepare grinding wheels. After dressing treatment, the grinding effect was finally achieved with a surface roughness of 1 nm. Zhao [24] proposed a chromium oxide (Cr_2_O_3_) gel abrasive tool to solve the problem of the agglomeration of abrasives and obtained a better experimental result than the traditional hot-pressing method.

However, there are few literature reports on the overall process from fine lapping to polishing for sapphire wafers. In this study, soft and hard mixed abrasives that could react with sapphire were selected to prepare the FAT, based on the principle of solid-phase reactions. The fixed-abrasive tools prepared in this study were used to carry out comparative experiments, and it is intended that the tool replace the traditional machining process of fine lapping and rough polishing. Research achievements can not only reduce the time needed for replacing equipment but also improve efficiency. In addition, it will save 1–3 h for the fine polishing stage and ultimately shorten the overall process’s time.

## 2. Experimental Materials and Methods

### 2.1. Preparation of the Fixed-Abrasive Tool

In the selection stage of abrasives, two basic criteria should be considered at the same time. First of all, solid-phase chemical reactions can occur on the surfaces of sapphire wafers under certain conditions. In addition, the hardness value of the abrasive must not be higher than the sapphire. By using HSC software and the DSC analysis method, thermodynamic analysis and reaction heat experiments were carried out to compare the Gibbs free energy change in different substances, with a temperature change implemented by Wang [25]. According to the result’s curve analysis, the substances that could have obvious chemical reactions with alumina include SiO_2_, ZnO, MgO, and Fe_3_O_4_. It is known from references [22,23,24] that Cr_2_O_3_ may be another good choice, and it has the highest Mohs hardness (8–8.5) among them.

It is necessary to take into account the micro-elasticity of the FAT after forming and curing processes and to ensure that the FAT has a suitable hardness. A type of silicone-modified phenolic resin–epoxy resin was selected as the binder in this study, which not only has low curing temperature requirements and small shrinkage after curing but also certain elasticity. At the same time, it could be easily dressed and meet the preparation conditions of FAT. In order to improve the bonding strength of FAT, KH-550 was selected as the coupling agent. Na_2_CO_3_ and NaHCO_3_ were selected as auxiliary materials in order to promote the occurrence of solid phase reactions and increase the material removal rate. In addition, with Na_2_CO_3_ and NaHCO_3_ particles dissolved in water, micropores could be formed on the FAT’s surface, which can be conducive to self-dressing and chip tolerance during processing.

**Figure 1:**
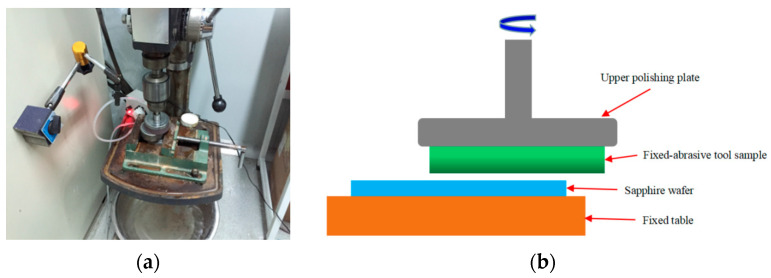
Preliminary experiment diagram: (**a**) physical diagram and (**b**) schematic diagram.

**Figure 2:**
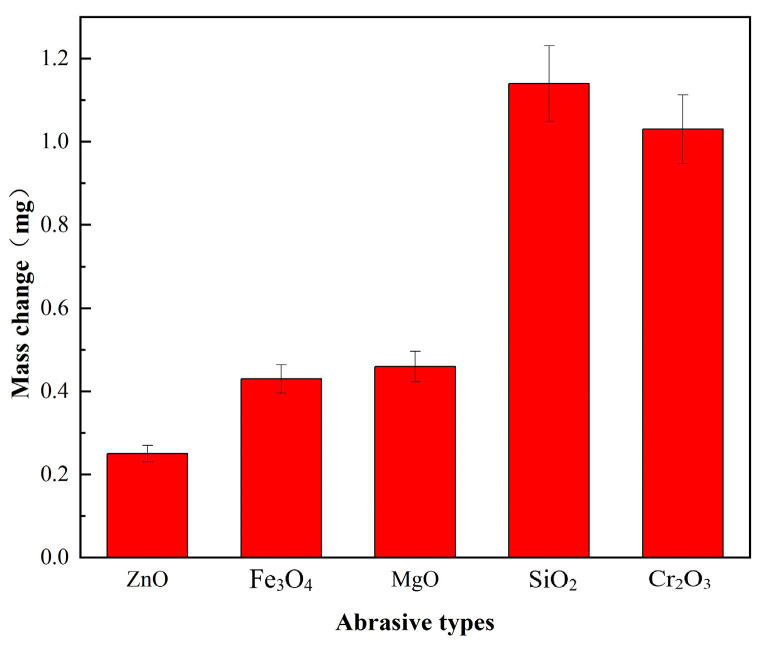
The mass change in the sapphire wafer before and after processing using different abrasive types.

A simply modified Xi-hu Z516A multi-functional desktop drilling machine was applied to conduct preliminary experiments in order to test the effectiveness of different types of abrasives. Sapphire wafers provided by Tiantong Company (China) were 2 inches in diameter with an initial thickness of 430 ± 5 μm, and it was settled on the fixed table. The FAT sample for testing was settled on the lower surface of the upper plate. The diameter of the sample tool was 60 mm, and the rotation speed of the upper plate was 210 rpm. The processing time was 20 min. A constant loading force was provided by the spring. The physical and schematic diagrams are shown in Figure 1. The effectiveness of abrasives was compared by measuring the mass change in the sapphire wafer before and after processing, and the result is shown in Figure 2. As shown in Figure 2, SiO_2_ and Cr_2_O_3_ obtained better material removal rates, even at 2 to 3 times higher efficiencies than other abrasives. Thus, these two types of abrasives were chosen as the application objects.

An STV Small V-type mixer was used to mix the abrasives and excipients. A hot forming press machine from Dazheng Technology Co., Ltd., Tengzhou, China, was used for molding. The LX-D Shore hardness tester was used to measure the hardness of the FAT. An RGM-4000 electronic universal testing machine was applied to test the compressive strength of the FAT. A KEYENCE VHX 7000 optical microscope and ZEISS ΣIGMA scanning electron microscope were used to observe the microstructure of the FAT’s surface.

**Figure 3:**
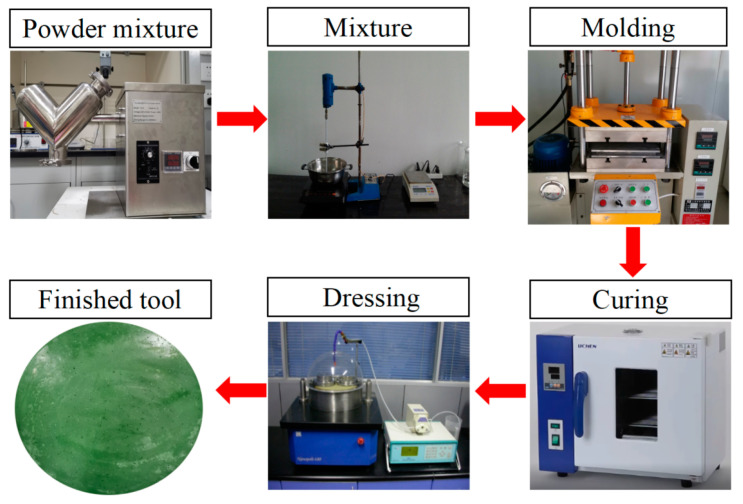
The main preparation process of the FAT.

Firstly, powders such as abrasives and auxiliary materials were mixed for 25 to 30 min to ensure uniform dispersion. Then, the powders in the previous step were mixed and stirred with the binder, wetting agent, and coupling agent in a sequence for 30 min. The casting and the hot-pressing methods were carried out and combined to achieve molding. The curing stage lasts for 1.5 to 2 h in a constant temperature oven at 85 degrees Celsius. After this stage, the fixed-abrasive tool was demolded and dressed. The specific preparation process of the FAT is shown in Figure 3.

Examining previous research results, it was found that large particle-size abrasives have better removal effects on sapphire materials than fine particle-size abrasives. Furthermore, [25] also confirmed this view. This study is mainly aimed at the processing of sapphire specimen in the stage of fine lapping and rough polishing; SiO_2_ and Cr_2_O_3_ abrasive sizes of 800#(19 μm), 1500#(10 μm), and 3000#(5 μm) were selected. The mass ratios of SiO_2_ and Cr_2_O_3_ abrasives were set as 0, 0.5, 1, 2, and positive infinity. The mass fraction of abrasives was selected as 45 wt%, 55 wt%, and 65 wt%. Evenly stirring fixed-abrasive tools with higher mass fractions was difficult under the condition of choosing 3000# SiO_2_ and Cr_2_O_3_ abrasive particles, and internal collapse or even no molding would occur in the later curing stage. We used 65 wt% as the upper limit of the abrasive mass fraction.

### 2.2. Lapping and Polishing Experiment

The UNIPOL-1200S automatic pressure polishing machine was used to carry out the processing experiment. A Kamoer F01A-STP smart small peristaltic pump was used to drip deionized water or polishing liquid. The surface of sapphire wafers was evaluated using scanning electron microscopy (SEM; ZEISS SIGMA, Carl Zeiss AG, Oberkochen, Germany) and an optical 3D surface profiler (SuperView W1, Chotest, Shenzhen, China). An electronic balance (Sartorius MSA225S, SSIL, Goettingen, Germany) was applied to measure the mass loss of the workpiece before and after processing. Polyurethane was used as the polishing pad during rough polishing and CMP.

In order to verify whether the prepared FATs have the potential to replace traditional fine lapping and rough polishing processes, the comparison experiment was designed using two methods. In this experiment, the undamaged surface was taken as the final evaluation standard. With respect to the first method, the traditional sapphire production process was adopted under laboratory conditions, which comprised copper plate polishing + rough polishing + chemical mechanical polishing (CMP). The other method was to use the prepared FAT for processing + CMP.

**Figure 4:**
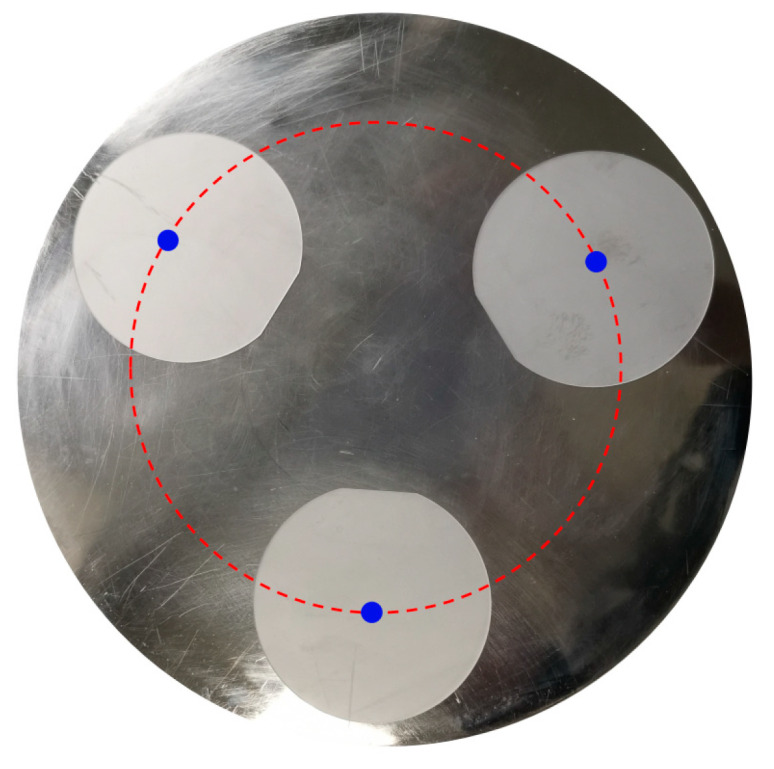
C-plane sapphire wafers on the carrier plate.

The processing parameters of copper plate polishing and rough polishing processes are shown in Table 1 and Table 2, respectively. The processing parameter of the FAT is shown in Table 3. The final CMP in both methods was carried out under the same processing parameters, which are listed in Table 4. The rotation speed of the loading plate and polishing plate was set at 60 rpm in all experiments. In order to ensure the authenticity and effectiveness of the data from each group of processing experiments, three pieces of C-plane sapphire wafers with similar surface quality were selected and distributed evenly on the carrier plate, as shown in Figure 4. The three centers of the workpieces are approximately distributed on a circle.

## 3. Results and Discussion

### 3.1. Compositional Optimization of FAT

The orthogonal test was designed to optimize the FAT composition. In this experiment, three factors (A stands for SiO_2_/Cr_2_O_3_ mass ratio, B stands for mass fraction, and C stands for particle size) and three levels (A1 = 0; A2 = 1; A3 = ∞; B1 = 45 wt%; B2 = 55 wt%; B3 = 65 wt%; C1 = 19 μm; C2 = 10 μm; C3 = 5 μm) were selected; thus, orthogonal table L_9_ was chosen.

In order to simplify the experiment, the same particle size was used for different types of abrasives. The specific experimental scheme and results are shown in Table 5. The material removal rate is converted using the weighing method. The material removal rate was calculated using Formula (1):

(1) $$MRR=\frac{\Delta m}{\rho \pi r^{2}t}\times 10^{7}$$

where *MRR* denotes the material removal rate, Δm denotes the mass change value of sapphire substrates, ρ denotes the density of sapphire substrates, ρ = 3.98 g/cm^3^, *r* denotes the radius of sapphire substrates, which equals 25.4 mm, and *t* is the processing time.

The analysis of the orthogonal experiment’s results is shown in Table 6. *K*_1_, *K*_2_, and *K*_3_ and the corresponding *k*_1_, *k*_2_, and *k*_3_ are the sum and mean values of each level, respectively. By comparing the range *R* of A, B, and C, we can conclude that the order of influencing factors is A > C > B. The mass ratio of SiO_2_/Cr_2_O_3_ has the greatest influence on the final material removal rate. From the *k* value of each factor, it can be observed that factor A has the largest value at *k*_2_; thus, A_2_ was the best. Similarly, B takes B_2_, and C takes C_2_; thus, the optimal combination is A2B2C2. The mass fraction of the abrasives is 55 wt%, and the particle size of SiO_2_ and Cr_2_O_3_ abrasives is selected as 10 μm.

**Figure 5:**
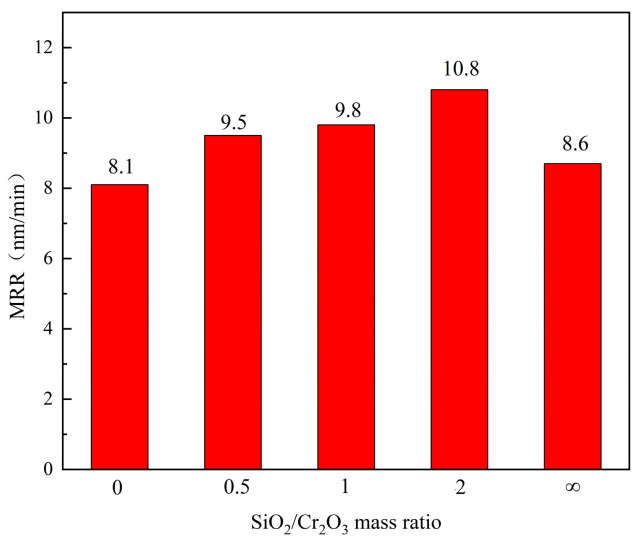
Influence of the SiO_2_/Cr_2_O_3_ mass ratio on the removal rate.

Aiming at further optimizing the abrasive mass ratio, the mass ratio of the SiO_2_ and Cr_2_O_3_ abrasive was set as 0, 0.5, 1, 2, and positive infinity. The influence of the SiO_2_/Cr_2_O_3_ mass ratio on the material removal rate is shown in Figure 5.

It can be concluded that when the mass ratio of SiO_2_/Cr_2_O_3_ is 2, the material removal rate is the highest, and the MRR of the mixed abrasive is higher than that of a single abrasive from Figure 5. It is known from reference [22] that sapphire can react with silicon oxide under certain conditions as described in Formulas (2) and (3). In [23], Wu speculated that sapphire and chromium oxide may chemically react, as shown in Formula (4).

3Al_2_O_3_ + 2SiO_2_ = 3Al_2_O_3_•2SiO_2_ (2)

Al_2_O_3_ + 2SiO_2_ + 2H_2_O = Al_2_Si_2_O_7_•2H_2_O (3)

Cr_2_O_3_ + Al_2_O_3_ = (Cr_x_Al_1−x_)_2_O_3_ (4)

Both SiO_2_ and Cr_2_O_3_ abrasives can react with sapphire in the solid phase. However, the condition for SiO_2_ to react with sapphire is easier than Cr_2_O_3_; thus, the MRR of a single SiO_2_ abrasive is higher than that of a single Cr_2_O_3_ because the hardness of Cr_2_O_3_ is 8~8.5, which is higher than SiO_2_. The energy generated by the friction between Cr_2_O_3_ and the surface of the workpiece is higher when in contact with the surface of the sapphire workpiece, which is conducive to the solid phase reaction between SiO_2_ and the sapphire. The mass ratio of SiO_2_/Cr_2_O_3_ is 2, and the material removal rate of the sapphire wafer is the highest. The final optimized formula of the FAT is shown in Table 7.

### 3.2. Surface Topography and Physical Properties of FAT

#### 3.2.1. Surface Topography of FAT

**Figure 6:**
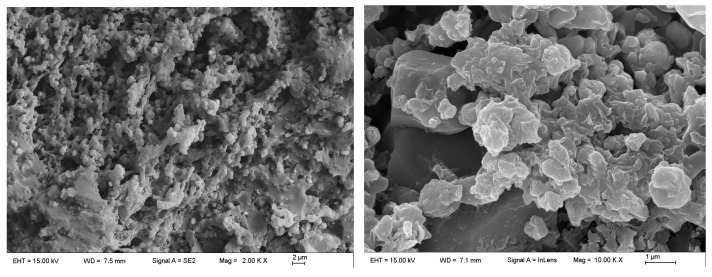
SEM topography of the dressed FAT surface at different magnifications.

The surface of FAT was dressed using a diamond dressing wheel. Figure 6 shows the SEM images of the surface morphology after dressing at different magnifications. Abrasive particles distributed on the surface and obvious pores can be clearly observed at 2.0K× and 10.0K× magnifications.

#### 3.2.2. Physical Properties of FAT

Test samples were prepared according to the mode’s size, with a diameter of 40 mm and a thickness of 6 mm. The hardness and compressive strength statistics are shown in Table 8 in terms of 9 points relative to the FAT with different compositions.

The less abrasive the mass fraction in the FAT, the higher the hardness of the fixed-abrasive tool. However, different abrasives with the same mass fraction have little difference in hardness. The changing rule of the compressive strength of FAT with different compositions is similar to that of hardness. The test results showed that the physical properties of the FAT sample meet the experimental requirements of this study.

### 3.3. Lapping and Polishing Experiment Results

#### 3.3.1. Traditional Sapphire Production Process

According to the preliminary studies of the research group, the efficiency of free abrasive processing may be the highest when the rotating speed is 60 rpm. Accelerating the rotational speed leads to an increase in centrifugal force and a decrease in the utilization rate of the polishing slurry.

(1) Copper plate polishing (fine lapping)

**Figure 7:**
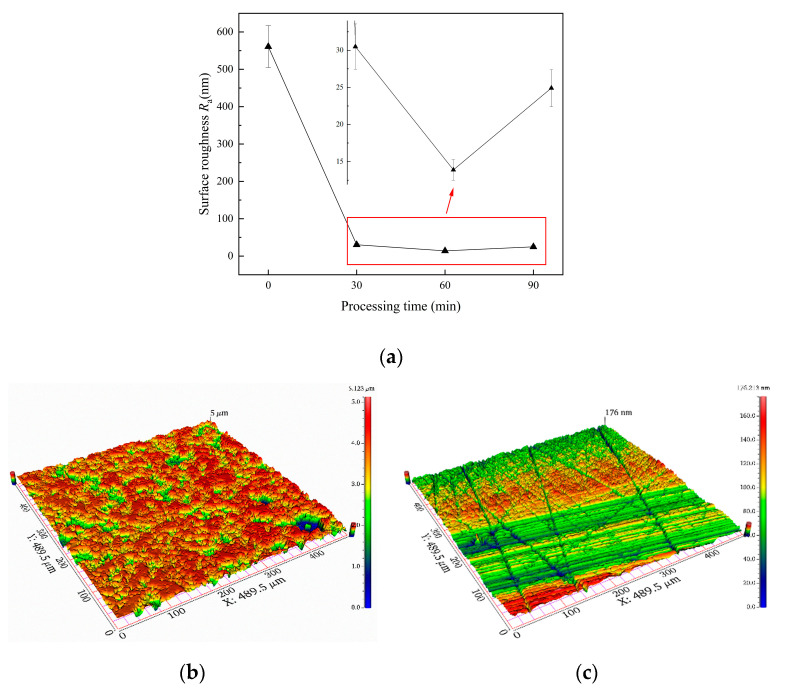
The variation in surface roughness at the copper plate polishing stage and 3D surface morphology: (**a**) variation in *R*_a_ with respect to the processing time (**b**) before processing and (**c**) after 60 min processing.

The surface roughness *R*_a_ of the sapphire wafer decreased from 560.9 ± 52.9 nm at the initial stage to 30.5 ± 2.2 nm at 30 min. After 60 min, the roughness decreased to 13.9 ± 1.2 nm. Then, 90 min later, the surface roughness *R*_a_ increased to 25.0 ± 2.1 nm. Therefore, the copper polishing time of 60 min may be the best. After repeating the experiments three times, the average surface roughness *R*_a_ was 15.2 ± 1.1 nm, and the material removal rate was 368.4 ± 28.9 nm/min after copper polishing for 60 min. Nevertheless, there were obvious scratches on the surface. Figure 7a shows the variation in surface roughness at the copper plate polishing stage with respect to processing times. Surface topographies before and after processing are shown in Figure 7b,c.

(2) Rough polishing

**Figure 8:**
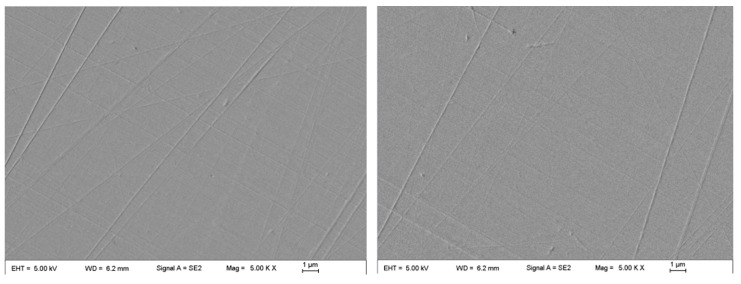
Typical SEM surface morphology in the two chosen areas after rough polishing.

After repeating the experiments three times, it was observed that the best surface roughness can be obtained after 60 min at this stage. After 90 min, the roughness increased, and the number of tiny scratches increased significantly. The average surface roughness *R*_a_ was 6.4 ± 0.9 nm, and the material removal rate was 12.8 ± 1.1 nm/min after 60 min of rough polishing. The surface morphology observed using SEM is shown in Figure 8.

(3) Fine polishing (CMP)

**Figure 9:**
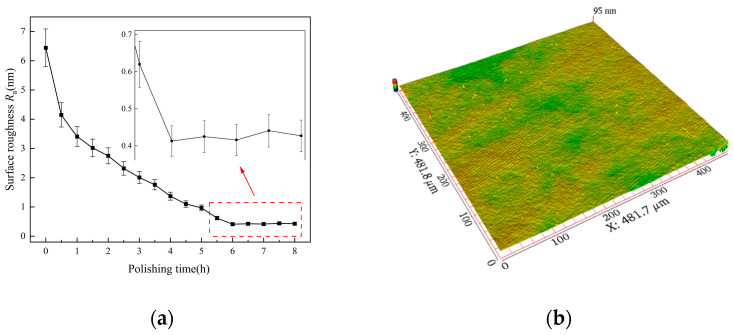
The surface quality after CMP: (**a**) variation in surface roughness and (**b**) surface topography.

The final process was fine polishing via the CMP method. Polyurethane pads with nano-sized particle silica sol were used in the final processing stage. The main purpose was to remove the scratches caused by the previous process and finally obtain a surface quality with no damage. Scratches gradually decreased with the increase in processing time. After 6 h, the scratches were completely removed, the average *R*_a_ was 0.4 ± 0.1 nm, and the material removal rate was 8.0 nm/min. After that, machining continued for 2 h. The results showed that the surface roughness had no obvious improvement, according to Figure 9a. Therefore, it can be considered that the best surface quality can be obtained after 6 h using CMP. The surface’s topography is shown in Figure 9b.

#### 3.3.2. Prepared FAT for Processing + CMP

(1) Prepared FAT for processing

**Figure 10:**
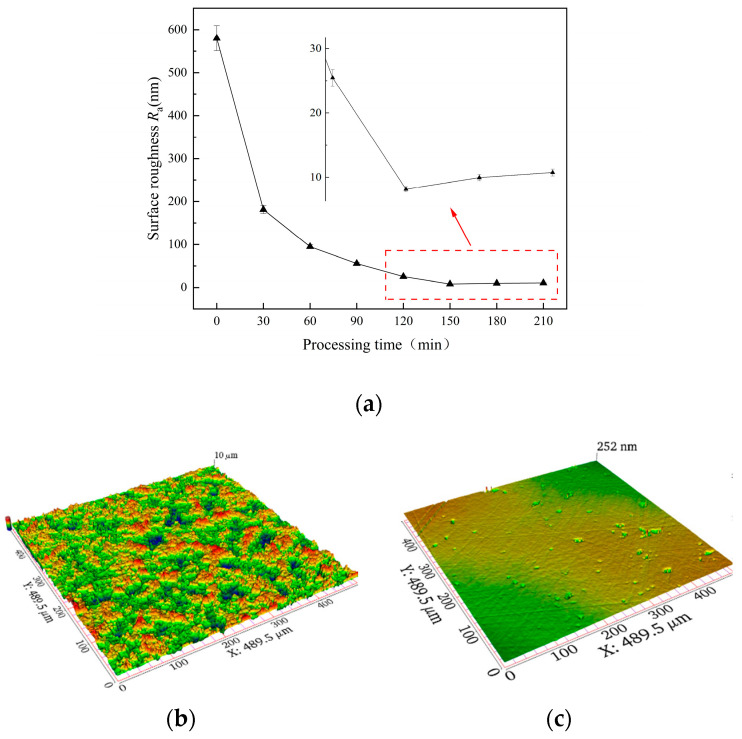
The variation in surface roughness using FAT and 3D surface morphology: (**a**) variation in *R*_a_ relative to processing time—(**b**) before processing and (**c**) after 150 min processing.

The surface roughness *R*_a_ of the sapphire wafer decreased from 580.4 ± 52.7 nm at the initial stage to 8.1 ± 0.7 nm at 150 min, which can be observed in Figure 10a. During this period, surface roughness *R*_a_ decreased with an increase in processing time. Due to this, roughness can deteriorate even further, and the surface quality will not be significantly improved. Therefore, the best time for FAT processing may be 150 min. After repeating experiments three times, the average surface roughness *R*_a_ was 8.0 ± 0.6 nm, and the material removal rate was 14.3 ± 1.2 nm/min. The three-dimensional surface morphology before and after FAT processing is shown in Figure 10b,c.

There were no obvious scratches on the surface of the workpiece, but there were some pits and slight machining marks. It could be supposed that the material removal mechanism of the sapphire wafer comprised the following: the surface of the workpiece reacted with the SiO_2_ and Cr_2_O_3_ abrasives according to Formulas (2)–(4). Both SiO_2_ and Cr_2_O_3_ have a lower hardness than sapphire. Via the mechanical action of friction and extrusion, a solid-phase chemical reaction occurs between the sapphire workpiece and SiO_2_ and Cr_2_O_3_ abrasive particles. Then, a metamorphic layer is formed. The hardness of the reaction layer is lower than that of abrasive particles. As a result, there were some pits and slight scratches on the left surface of the workpiece.

(2) Fine polishing (CMP)

**Figure 11:**
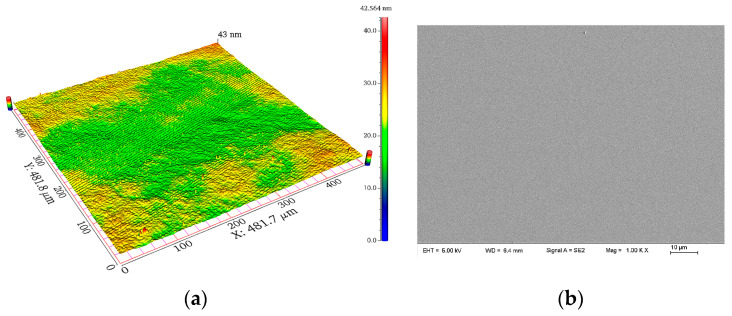
The surface quality after CMP: (**a**) 3D morphology and (**b**) SEM surface topography.

At this stage, all conditions were the same as those in the previous CMP experiment, except for the different processing methods used on the workpiece. With the increase in processing time, the surface quality was gradually improved. After 4 h, the pits and slight scratches were completely removed, the average *R*_a_ was 0.4 ± 0.1 nm, and the material removal rate was 7.6 ± 0.8 nm/min. After that, machining was continued for 1.5 h. The results showed that the surface roughness had no obvious improvements. Therefore, it was considered that the best surface quality could be obtained after 4 h using CMP with a final *R*_a_ of 0.4 ± 0.1 nm. The surface’s topography is shown in Figure 11.

#### 3.3.3. Comparison of the Two Methods

This experiment does not take the removal rate as the evaluation index, but it takes the final undamaged surface and the lowest surface roughness as the goal of the analysis. The total time of the first method (traditional sapphire production process) requires 8 h to achieve the best surface quality. Among them, the copper plate polishing (fine lapping) stage requires 1 h, the rough polishing stage requires 1 h, and CMP requires 6 h. The reason for the long CMP time is that a diamond abrasive was used for fine lapping and rough polishing. Diamond abrasives can cause a large number of scratches on the surface of workpieces, which require a long period of removal using CMP. The other method (FAT processing and CMP) requires 6.5 h to achieve the best surface quality. FAT processing requires 2.5 h, and CMP requires 4 h. Thus, FAT processing combined with CMP costs less time compared to the first method. Furthermore, the polishing plate’s rotation speed could be increased in the FAT processing experiment because FAT is almost unaffected by centrifugation. Higher processing efficiency could be obtained, and less time could be used by increasing the rotation speed of the polishing plate.

## 4. Conclusions

(1) Using the design of the pre-experiment and orthogonal experiment, the optimal FAT composition under the experimental data was determined. The mass ratio of abrasives was 55 wt%, and the mass ratio of SiO_2_/Cr_2_O_3_ was 2.
(2) Different abrasives with the same mass fraction have little difference in terms of hardness. The less abrasive the mass fraction in FAT, the higher the hardness. The changing rule of the compressive strength of FAT with different compositions is similar to that of hardness.
(3) Better results can be obtained in sapphire wafer processing using the prepared FAT. The final average surface roughness *R*_a_ was 8.1 ± 0.7 nm, and the material removal rate was 14.3 ± 0.2 nm/min.
(4) FAT processing combined with CMP is shortened by 1.5 h compared to the traditional sapphire production process in terms of achieving the best surface quality. This research achievement may have the potential to shorten the processing time of the sapphire polishing industry.

## Figures and Tables

**Table 1:** Processing parameter of the copper plate polishing process.

Experimental Condition	Parameter
polishing plate	ϕ300 mm resin copper plate
rotation speed of the polishing plate	60 rpm
loading	4 kg
processing interval	30 min
slurry	5 μm 0.6 wt% diamond

**Table 2:** Processing parameter of the rough polishing process.

Experimental Condition	Parameter
polishing plate	ϕ300 mm polyurethane pad
rotation speed of the polishing plate	60 rpm
loading	4 kg
processing interval	30 min
slurry	1.3 μm 5 wt% diamond

**Table 3:** Processing parameter of FAT processing.

Experimental Condition	Parameter
polishing plate	prepared FAT
rotation speed of the polishing plate	60 rpm
loading	4 kg
processing interval	30 min
slurry	deionized water

**Table 4:** Processing parameter of CMP.

Experimental Condition	Parameter
rotation speed of the polishing plate	60 rpm
loading	4 kg
processing interval	30 min
slurry	5 wt% 80~120 nm silica sol
pH of slurry	10.7

**Table 5:** Orthogonal experimental scheme and results.

Number	A	B	C	MRR (nm/min)
	1	2	3	
1	A1	B1	C1	8.3
2	A1	B2	C2	10.2
3	A1	B3	C3	7.9
4	A2	B1	C2	11.6
5	A2	B2	C3	9.4
6	A2	B3	C1	10.8
7	A3	B1	C3	7.3
8	A3	B2	C1	8.7
9	A3	B3	C2	9.3

**Table 6:** Analysis of the orthogonal experiment’s results.

Results	A	B	C
	1	2	3
*K*_1_	26.4	27.2	27.8
*K*_2_	31.8	28.3	31.1
*K*_3_	25.3	28	24.6
*k*_1_	8.8	9.1	9.3
*k*_2_	10.6	9.4	10.3
*k*_3_	8.4	9.3	8.2
*R*	2.2	0.2	2.1

**Table 7:** The final optimized composition of the FAT.

Type	Abrasives	Binding Agent	Auxiliary Materials	Coupling Agent
ingredient	SiO_2_ and Cr_2_O_3_	modified resin	NaHCO_3_ and Na_2_CO_3_	KH-550
mass ratio	55 wt%	36 wt%	8 wt%	1 wt%

**Table 8:** Hardness and compressive strength statistics with different compositions.

Different Compositions	Mean Hardness	Mean Compressive Strength (MPa)
45 wt% SiO_2_	84	114.54
45 wt% Cr_2_O_3_	86	113.27
45 wt% SiO_2_ and Cr_2_O_3_	85	109.89
55 wt% SiO_2_	76	101.06
55 wt% Cr_2_O_3_	78	100.24
55 wt% SiO_2_ and Cr_2_O_3_	77	100.98
65 wt% SiO_2_	69	97.24
65 wt% Cr_2_O_3_	72	95.03
65 wt% SiO_2_ and Cr_2_O_3_	70	96.11

## Data Availability

The data presented in this study are available on request from the corresponding author. The data are not publicly available due to the need for further research.
